# Supplementary material for: Functional Identification of Arthrinium phaeospermum Effectors Related to Bambusa pervariabilis × Dendrocalamopsis grandis Shoot Blight
Source: Biomolecules. 2022 Sep 8;12(9):1264. doi: 10.3390/biom12091264 (PMC9496123; doi:10.3390/biom12091264)
Supplement: Supplementary file 1 [file biomolecules-12-01264-s001.zip › biomolecules-1874718-supplementary/Supplementary materials/Table S1-S4.pdf]

Table S1. Fluorescent quantitative PCR primers for *B. pervariabilis×D. grandis.*

iD	Gene name	Primers
50323_c4_g3	*ADF3*	GGTAGCAATCCAAGCACGAACA, CACGCCTCCTAAATCCTAATCAC
45871_c1_g2	*PE cDNA*	TTCGGATGGAAGCAAGGAGA, CAGATGTAACAGCGAGCAGGGT
49306_c2_g1	*ATSTP*	GCGTCCAACACTACAGCTCGAT, CGCCGTGATAAACTACAAGATGC
36800_c1_g1	*SIP*	GGTTGCCGTGAATGAGGTCC, GGCTCAGTAGCGGTGAAGGA
33659_c0_g3	*2,3-BIPM*	TCGTGTCAGATTCCCGAAGATT, GGTGCGTATGCCATTGTGC
46718_c1_g2	*PPP 2C8*	CTGAGTTTGCTGGGATGTTGTAGA, GCCGCTTCGGTCGTAATGT
46718_c1_g1	*PPP 2C*	TCTGCAATGCCATGCTCTGTG, GAGGTGGCTGTCACTTCTGTTCTT
17335_c0_g1	*AD*	GCAGCACTGTCCGTGGTTGA, CTTATTGGCCGCACCATCAT
56115_c2_g1	*HVSV*	GATTGCCAAACCATACCCATACA, CTGGAGAACAAACCGTGGATG
86830_c0_g1	*ARFLP*	CCGATGTGGGAACGGTATTG, GTTACCAAGCACGAGCAAAGG
44947_c1_g1	*Hpp*	TGGAGGAAGGAAACATAGAGCAC, GCAAGAAGCCAAGGAAGCAA
38053_c0_g1	TFHBP1a	GGCGAACAGTGGTGAACGAA, TGCCTCCAAGAAACCAATCAAG
48578_c1_g2	TFMYB48	CCTCCCAAACAAACAATGCC, TGGTGCTGATTGATAAGGATGG

Table S2. Fluorescent quantitative PCR primers for *A. phaeospermum.*

iD	gene name	Primers
AP-Z1311765	TLP1	CCTAACATCCACCAGAACAACAAG, CAGGCTGCCAAAGTAGTCGG
AP-Z1313924	FDLP	GCCCACTCAATCCTCAGTATCAC, CCTTGAGAAGCTTTGGCGC
AP-Z1316075	CDCP	ACAACAACAACTACGACCAAGGC, GCGTGCTGTAATAAGACGAGGC
AP-Z1300511	SH	CGTCGAACAGACAGAAGGTGGA, AAGAGTGATTGCGGACAGCG
AP-Z1300532	TB6H	ACGGTTGCCCTCGCCTTTA, GCTTTGCTGCCGGGAATCT
AP-Z1300714	EP1	GAGCAAGAGCACGCCAGGTT, TTAGCCTCCGCCTTCTCCTC
AP-Z1301399	CWPP	AACTCCAACTGCACCGCCA, ACGGGTTCCATGAAGTGCTGT
AP-Z1301547	CS	CCGGAACCACCATGCAATAT, ACAATCGGGAAGAGGAAGACG
AP-Z1301822	AR	TGGAAGGGACCAAGTTCAGCA, TCCCACAACACGACCTGACG
AP-Z1301741	EHLM	TTGTTCCAGATGCCAATGCG, GCGAGCTGTATCGGCGTGTA

Table S3. Primers for constructing and detecting pGR107-ApCEs recombinant vector.

ID	Primers Sequence
pGR-ApCE12-F	CAGCACCAGCTAGCATCGAT ATGCATACCAAATGTTCTTTCTGG
pGR-ApCE12-R	CGGTCGACCCGGGAATCGAT CTATAAGAATGTTTCTACCCGCGG
pGR-ApCE22-F	CAGCACCAGCTAGCATCGAT ATGAAGTACACCGCGATCGC
pGR-ApCE22-R	CGGTCGACCCGGGAATCGAT TTAAAGGACCATAGCCATAAGACC
pGR-ApCE28-F	CAGCACCAGCTAGCATCGAT ATGAAGTACTCACTCGCCCTCATC
pGR-ApCE28-R	CGGTCGACCCGGGAATCGAT CTAGAGCCCACCAAGAAGACCG
ApCE12	GTGTGGCAAGGTTGATATGTCG, AGGCCCTCGAACTTGACAGAC
ApCE22	ACTGGTATTGCTTCCGCCG, CCATAGCCATAAGACCAACAGCA
ApCE28	AGCCAAAGCCAAGCGATGC, CTAGAGCCCACCAAGAAGACCG
pGR107-F/R	GTGGTAACAATCATAGCAGTCA, AGTTGACCCTATGGGCTGTGTT

Note：It represents a sequence complementary of the PGR-107 vector ClaI enzyme digestion site upstream. It represents a sequence complementary of the PGR-107 vector ClaI enzyme digestion site downstream.

Table S4. ApCE12 and ApCE22 gene knockout and complement experimental primers.

Primier name	Primer sequence 5′-3′
ApCE12-5-F	TGGGATATGTCACATGCCTGG
ApCE12-5-R	AGTTCAGGCTTTTTCATATCTGCACAGCAAATTGGTTCGA
ApCE12-3-F	CGAGGGCAAAGGAATAGAGTTCTCAACTCCTAGTATTGCCTCG
ApCE12-3-R	TGGCGTCATTCAGCAAACC
ApCE22-5-F	TGCGGAAGCTGTTTGGATG
ApCE22-5-R	AGTTCAGGCTTTTTCATATCGCGTTTTGAACGTTTTGCGA
ApCE22-3-F	CGAGGGCAAAGGAATAGAGTCCGAATGGAAGAACCAACAA
ApCE22-3-R	CGGCAGGACAACAGCTACA
KanMx-F	CAGCTGAAGCTTCGTACGC
KanMx-R	GCATAGGCCACTAGTGGATCTG
ApCE12-F	ATGCATACCAAATGTTCTTTCTGG
ApCE12-R	CTATAAGAATGTTTCTACCCGCGG
ApCE12-R+KanMx	GCGTACGAAGCTTCAGCTGCTATAAGAATGTTTCTACCCGCGG
ApCE22-F	ATGAAGTACACCGCGATCGC
ApCE22-R	TTAAAGGACCATAGCCATAAGACC
ApCE22-R+KanMx	GCGTACGAAGCTTCAGCTGTTAAAGGACCATAGCCATAAGACC
Hph-F	GATATGAAAAAGCCTGAACT
Hph-R	ACTCTATTCCTTTGCCCTCG
ApCE12＋KanMX-5-F	TGGGATATGTCACATGCCTGG
ApCE12＋KanMX-5-R	CCAGAAAGAACATTTGGTATGCATTGCACAGCAAATTGGTTCGA
ApCE12＋KanMX-3-F	CAGATCCACTAGTGGCCTATGCTCTCAACTCCTAGTATTGCCTCG
ApCE12＋KanMX-3-R	TGGCGTCATTCAGCAAACC
ApCE22＋KanMX-5-F	TGCGGAAGCTGTTTGGATG
ApCE22＋KanMX-5-R	GCGATCGCGGTGTACTTCATGCGTTTTGAACGTTTTGCGA
ApCE22＋KanMX-3-F	CAGATCCACTAGTGGCCTATGCCCGAATGGAAGAACCAACAA
ApCE22＋KanMX-3-R	TGGCGTCATTCAGCAAACC
ApCE12-5+hph-F/R	ATGCATACCAAATGTTCTTTCTGG
ApCE12-5+hph-R	ACTCTATTCCTTTGCCCTCG
ApCE22-5+hph-F	ATGAAGTACACCGCGATCGC
ApCE22-5+hph-R	ACTCTATTCCTTTGCCCTCG
ApCE12-5+KanMX-F	TGGGATATGTCACATGCCTGG
ApCE12-5+KanMX-R	GCATAGGCCACTAGTGGATCTG
ApCE22-3+KanMX-F	TGCGGAAGCTGTTTGGATG
ApCE22-3+KanMX-R	GCATAGGCCACTAGTGGATCTG
ApCE12+KanMx-F	ATGCATACCAAATGTTCTTTCTGG
ApCE12+KanMx-R	GCATAGGCCACTAGTGGATCTG
ApCE22＋KanMX-F	ATGAAGTACACCGCGATCGC
ApCE22＋KanMX-R	GCATAGGCCACTAGTGGATCTG
ApCE12-5+hph-F	TGGGATATGTCACATGCCTGG
ApCE12-5+hph-R	ACTCTATTCCTTTGCCCTCG
ApCE22-5+hph-F	TGCGGAAGCTGTTTGGATG
ApCE22-5+hph-R	ACTCTATTCCTTTGCCCTCG

Note: It represents a sequence complementary to hph-F. It represents a sequence complementary to hph-R. It represents a sequence complementary to KanMx-F. It represents a sequence complementary to ApCE12-F. It represents a sequence complementary to KanMx-R. It represents a sequence complementary to ApCE22-F.
