# Supplementary figures and images for: Functional Identification of Arthrinium phaeospermum Effectors Related to Bambusa pervariabilis × Dendrocalamopsis grandis Shoot Blight
Source: Biomolecules. 2022 Sep 8;12(9):1264. doi: 10.3390/biom12091264 (PMC9496123; doi:10.3390/biom12091264)

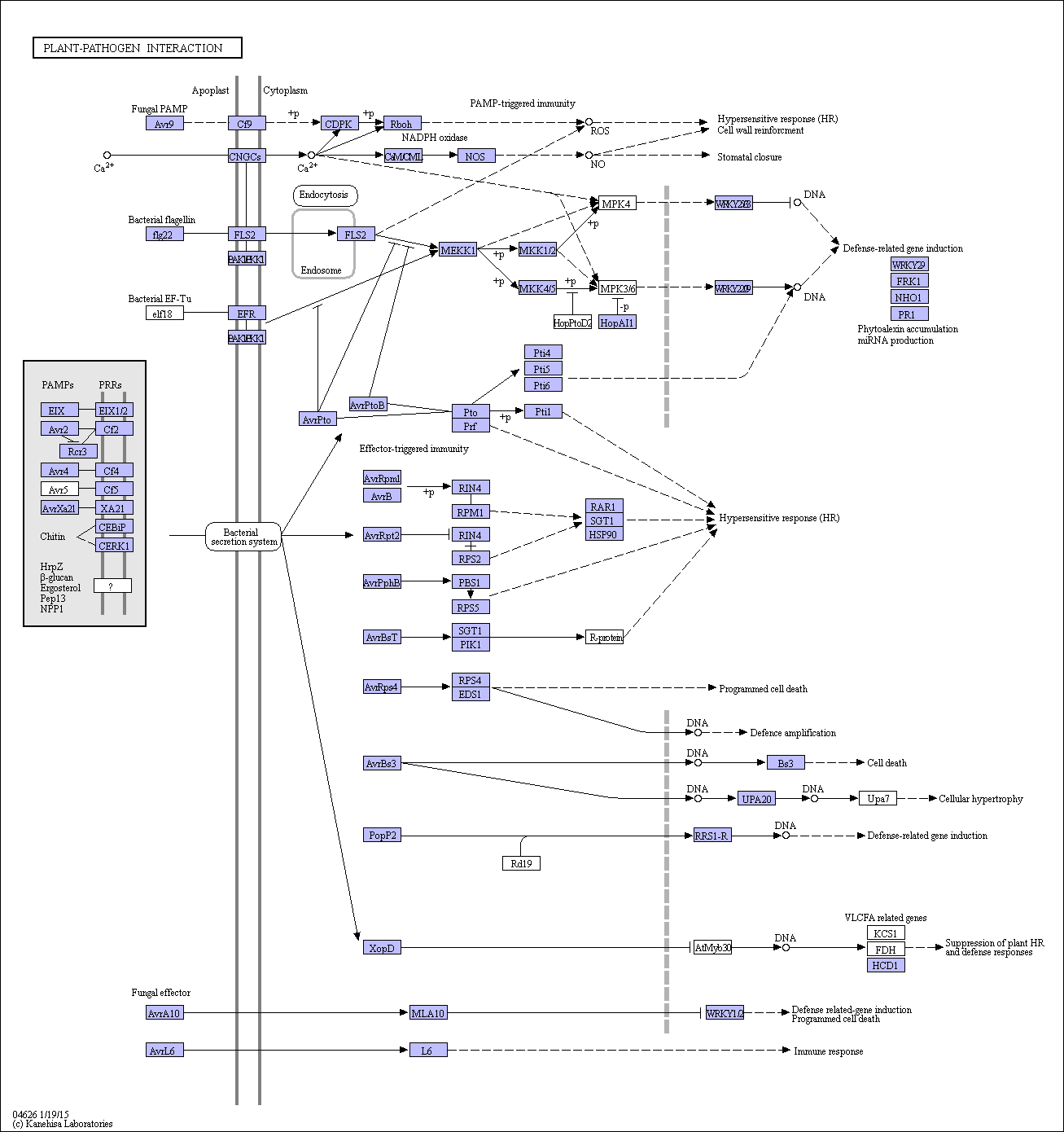

Supplement: Supplementary file 1 [file biomolecules-12-01264-s001.zip › biomolecules-1874718-supplementary/Supplementary materials/Figure S1.jpg]

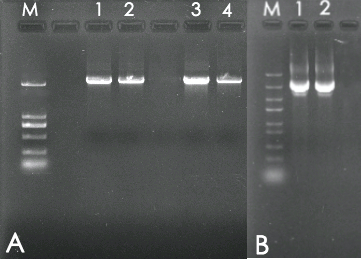

Supplement: Supplementary file 1 [file biomolecules-12-01264-s001.zip › biomolecules-1874718-supplementary/Supplementary materials/Figure S2.jpg]

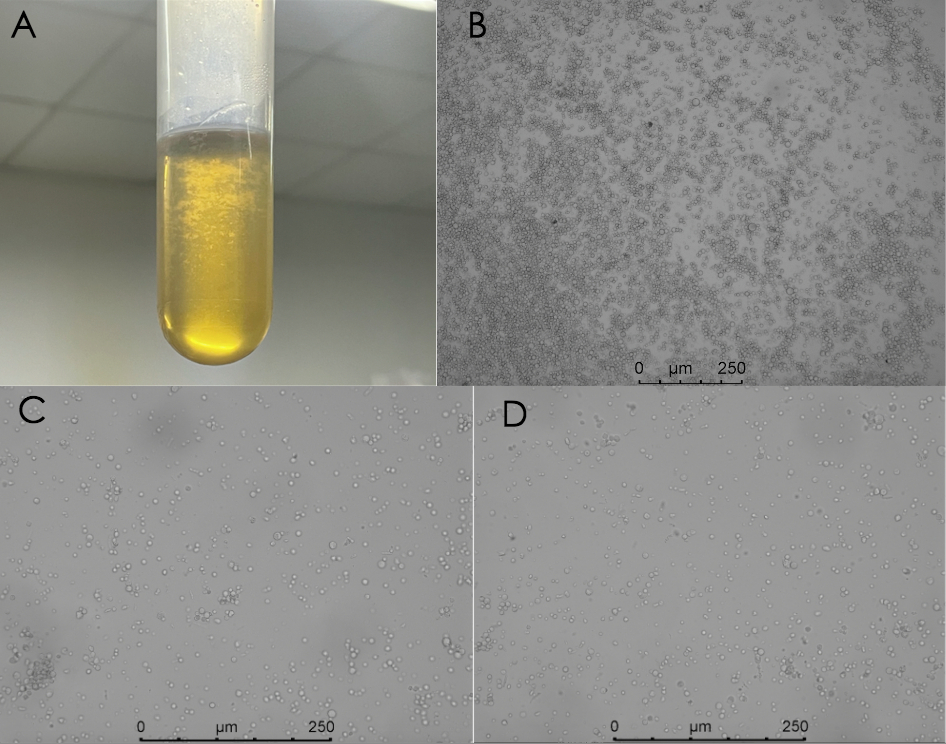

Supplement: Supplementary file 1 [file biomolecules-12-01264-s001.zip › biomolecules-1874718-supplementary/Supplementary materials/Figure S3.jpg]

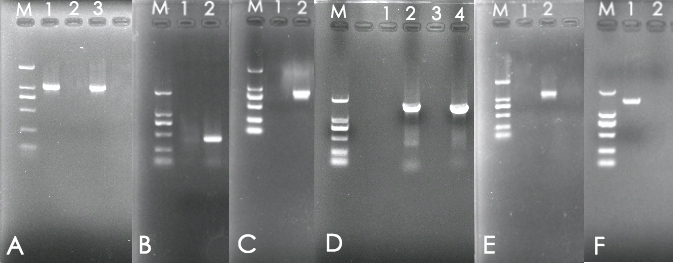

Supplement: Supplementary file 1 [file biomolecules-12-01264-s001.zip › biomolecules-1874718-supplementary/Supplementary materials/Figure S4.jpg]
